# Use of MRI fusion second-look ultrasound in breast cancer: can MRI US fusion reduce the need for MRI-guided biopsy?

**DOI:** 10.1186/bcr3253

**Published:** 2012-11-09

**Authors:** SE McWilliams

**Affiliations:** 1 Guy's and St Thomas' NHS Foundation Trust, London, UK

## Introduction

Breast MRI is being increasingly used in breast cancer to look for extent of disease, in high-risk screening and in the dense breast. Frequently incidental lesions are detected on MRI that require second-look ultrasound or stereotactic biopsy. Incidental MRI-detected lesions may be occult on conventional imaging and require MRI-guided biopsy. We describe our experiences with US MRI fusion to try and reduce the need for MRI-guided biopsy.

## Methods

At our institution we have introduced an MRI breast biopsy service which is time consuming and expensive. We looked at 10 patients with MRI US fusion technology on our new Hitachi US MRI scanner to see whether lesions were easier to identify combining the MRI and US images, enabling US biopsy to be performed.

## Results

Ten patients with a known breast cancer had a further incidental lesion seen on MRI. The patients had an additional supine series on contrast MRI images in addition to the standard prone protocol. The supine images were loaded on the US machine and enabled confident detection of the lesion on US in nine out of 10 patients.

## Conclusion

Using MRI US fusion with one additional MRI series of supine images reduces the need for MRI-guided biopsy enabling US biopsy to be performed, which is cheaper, quicker and more patient acceptable.

